# Integrative analysis of bulk and single-cell transcriptomic data reveals novel insights into lipid metabolism and prognostic factors in hepatocellular carcinoma

**DOI:** 10.1007/s12672-024-01487-y

**Published:** 2024-10-25

**Authors:** Feiyu Qi, Guiming Zha, Yanfang Zhang, Sihua Liu, Yuhang Yang, Wanliang Sun, Dongdong Wang, Zhong Liu, Zheng Lu, Dengyong Zhang

**Affiliations:** 1 Department of General Surgery, The First Affiliated Hospital of Bengbu Medical College, No.287 Chang Huai Road, Bengbu, 233000 Anhui China; 2 Department of Chest Surgery, The First Affiliated Hospital of Bengbu Medical College, Bengbu, 233000 Anhui China

**Keywords:** Hepatocellular carcinoma, HCC, Transcriptome analysis, Single-cell sequencing, Prognostic factors

## Abstract

Hepatocellular carcinoma (HCC) is associated with high mortality rate. This study investigated the status of lipid metabolism-related genes in HCC. Bulk transcriptomic and single-cell sequencing data for HCC were retrieved from public databases. The single-cell sequencing data was subjected to dimensionality reduction, which facilitated the annotation of distinct cell subpopulations and marker gene expression analysis within each subpopulation. Genes associated with lipid metabolism in liver cells were identified, and a machine-learning model was developed using the bulk transcriptomic data randomly partitioned into training and validation sets. The efficacy of the model was validated using these two sets. A multifactorial Cox analysis on the model genes combined with clinical features, led to the identification of age, HMGCS2, HNRNPU, and RAN as independent prognostic factors, which were included in the nomogram model construction and validation. A weighted gene co-expression analysis of all genes of the bulk transcriptome samples revealed the correlation between gene modules and risk score. Genes with cor > 0.4 in the highest-expressing module were selected for Gene Ontology and Kyoto Encyclopedia of Genes and Genomes functional enrichment analysis. Immune-related analysis was conducted based on seven algorithms for immune cell infiltration prediction. For the genes in the nomogram model, the expression in clinical pathological factors was also analyzed. The drug sensitivity analysis offered a reference for the selection of targeting drugs. This investigation provides novel insights and a theoretical basis for the prognosis, treatment, and pharmaceutical advancements for patients diagnosed with HCC.

## Introduction

Liver cancer is the sixth most prevalent cancerworldwide and the fourth leading cause of cancer-related deaths globally [1]. In 2020, there were 905,677 new cases of liver cancer, amounting to a rate of 4.7%, and 830,180 fatalities, indicating amortality rate of 8.3% [2]. Despite the decline in incidence rates, liver cancer remains a significant global health concern, and estimated to increase to one million cases by 2025 [3]. HCC is the predominant type of liver cancer, representing approximately 90% of cases [4]. Its development is primarily attributed to chronic liver disease and various factors such as infections with hepatitis B virus and hepatitis C virus, alcohol consumption, genetic mutations, and non-alcoholic fatty liver disease [1]. Key risk factors for HCC vary by region; in most high-risk areas for HCC, such as China, South Korea, and sub-Saharan Africa,and the key determinants arechronic hepatitis B virus infection and exposure to aflatoxins [2]. Gender differences may also be a confounding factor, with nearly 2 to 8 times higherincidence rates in males than females in both low and high-incidence regions [5]. Currently, various imaging techniques, such asultrasound, computed tomography, magnetic resonance imaging, and nuclear medicine, are employed for HCCdiagnosis. While liver resection or transplantation are the optimal treatments for these cases, only 20% of HCC cases are amenable to surgical intervention. Patients unsuitable for surgery requireNon-surgical interventions, such as radiofrequency ablation, transarterial chemoembolization, percutaneous microwave coagulation therapy, percutaneous ethanol injection, cryoablation, laser ablation, and acetic acid injection [6].

Despite progress in treatment modalities like chemotherapy and immunotherapy, the prognosis for liver cancer remains bleak, andcases in their advanced stage display less than 12% 5-year overall survival (OS) rate. In the early-stage the progression is frequently subtle, leading to delayed diagnosis and unfavorable prognoses,while late-stage instances demonstrate metastatic potential [7]. Although certain treatments, such as transarterial chemoembolizationare potentiallyeffectivein mid-stage liver cancer, managing late-stage cases of liver cancer remain challenging [8]. Early detection remains the only hope for a cure forpatients with HCC, underscoring the crucialimportance of effective screening [9]. Therefore, the mechanisms driving initiation and progression of HCC, particularly those associated with heightened rates of metastasis and recurrence,must be thoroughly explored. The discovery of keybiomarkers and investigation of key target genes are essential for enhancing HCCtreatment, diagnosis, and prognostic accuracy.

Several studies have investigated the mechanisms of specific gene families associated with HCC and have attempted constructingprognostic models [10, 11]. Research has also indicated an association between genes involved in lipid metabolism and HCC. For instance, the response of the mechanical reaction pathway dependent on stearoyl-CoA desaturase 1 enzymeto increased matrix stiffness (biomechanical signals in the tumor microenvironment) has been linked to enhanced HCC invasion and metastasis through lipid metabolism reprogramming [12]. Nevertheless, the predictive significance of genes involved in lipid metabolism towardimmune efficacy remains yet to be unraveled.

We downloaded and analyzed bulk transcriptome sequencing data and single-cell sequencing data of HCC from the public databases The Cancer Genome Atlas (TCGA) and Gene Expression Omnibus (GEO).We performed dimensionality reduction annotated cell subtypes on single cells and analyzed the expression of marker genesin each subtype. We utilized Gene Set Enrichment Analysis (GSEA) to acquire the cellular response to the lipid gene set and selected the overlap of this gene set with marker genes of the hepatocyte subtype through univariate Cox analysis to screen for genes related to lipid metabolism in liver cells. Subsequently, we classified bulk transcriptome data randomly into validation and training sets, constructed machine learning models, and employed these two cohorts to validate the models. We performed multifactorial Cox analysis by combining model genes with clinical features to select independent prognostic factors, using parts with p < 0.05 to construct and validate the Nomogram model. We also performed weighted gene co-expression analysis on all samples in the bulk transcriptometo observe the correlation between gene modules and Risk Score, and selected genes with cor > 0.4 in the highest expression module for Gene Ontology (GO) and Kyoto Encyclopedia of Genes and Genomes (KEGG) analysis. Further, immune-associated analysis was performed based on seven algorithms predicting immune cell infiltration. We also analyzed genes in the Nomogram model and their expression in clinic-pathological factors. Data for the selection of targeted drugs was obtained through drug sensitivity analysis. Our study thus provides new insights and a theoretical basis for prognostic judgment and drug development in HCC.

## Material and methods

### Data acquisitionand preprocessing

Corresponding clinical information for HCC and bulk transcriptomic data were obtained from the TCGA public database (https://portal.gdc.cancer.gov/). Additionally, single-cell sequencing data for liver cancer was retrieved from the GEO database (accession number GSE112271; https://www.ncbi.nlm.nih.gov/geo/). Samples lacking relevant information were excluded from the analysis. When a gene was represented by multiple rows in the expression matrix, the average data from these rows was utilized. Patients from the TCGA-HCC dataset were randomly classified into two equally sized groups: the training cohort and the testing cohort. Furthermore, the gene set associated with cellular response to lipids was obtained from the GSEA database (https://www.gsea-msigdb.org/gsea). All publicly available databases employed in this study allow unrestricted access and utilization of data, with no prerequisite of additional ethical approval. Data acquisition and analysis procedures were conducted by adhering to relevant guidelines and regulations.

### Single-cell sequencing analysis

Single-cell RNA sequencing data were analzed by utilizing the Seuart package. The accuracy and reliability of subsequent analyses were obtained upon quality control and data cleaning. Our criteria for data inclusion were as follows: percent.mt < 10, nCount_RNA > 1000, and nFeature_RNA between 100 and 5000. Subsequently, UMAP was employed for the dimensionality reduction of the single-cell data. Cell subtypes were annotated based on specific markers for each subtype, followed by visualization of the results. We generated heatmaps to elucidate the patterns of expression of marker genes within individual cell subtypes. This comprehensive assessment approach enabled the identification and characterization of distinct cellular subtypes, facilitating an enhanced understanding of the single-cell landscape in our study.

### Screening of hepatocyte lipid metabolism-associated genes

We acquired the “cellular response to lipid” gene set from GSEA and integrated this gene set with marker genes specific to the hepatocyte subtype. Subsequently, single-variable Cox analysis was conducted to screen for intersected genes and the results were visualized using a forest plot. Comprehensive scores were assigned to each sample based on the intersected genes employing the Single-sample GSEA (ssGSEA) algorithm. Based on the median score, samples were classified into high- and low-risk cohorts, followed by KM curve analysis to depict differences in OS, progression-free interval (PFI), and disease-specific survival (DSS) between these groups. Additionally, Gene Ontology (GO) and Kyoto Encyclopedia of Genes and Genomes (KEGG) analyses were performed for genes selected through single-variable Cox analysis to identify enriched functional pathways, the results were visualized using bubble plots. This integrated approach facilitated the discovery of important genes linked to hepatocyte lipid metabolism, offering insights into their potential contributions to liver function and disease progression.

### Construction and validation of machine learning prognostic models

We randomly divided the TCGA-HCC gene set into equal-sized training and testing cohorts. We devised a collection of 101 varied machine learning models, a combination of machine learning algorithms, including CoxBoost, stepwise Cox, Enet, RSF, Ridge, Lasso, SuperPC, plsRcox, GBM, and survivalSVM. These models were assessed in both two sets to obtain the respective C-index and average C-index values. According to the criteria for determining the optimal model combination, the C-index and average C-index of each set must not be less than 0.6. Subsequently, we selected the combination of machine learning algorithms with the highest average C-index to establish our predictive model. To estimate the risk score, we initially multiplied the gene expression levels by their corresponding coefficients and summed the outcome values of all model genes, as depicted below:

$$\text{Risk score}=\sum_{i=1}^{n}[{Exp}_{{gene}_{i}}*{\beta }_{i}]$$

here, ${Exp}_{{gene}_{i}}$ represents the expression level of the model gene i, and ${\beta }_{i}$ denotes the coefficient corresponding to the model gene. We assessed the correlations of the expression of model genes and presented them using chord diagrams. Subsequently, multifactor Cox analysis was conducted to identify independent prognostic factors by combining model genes with clinical features, such as gender, age, stage, and grade. Kaplan–Meier (KM) curves were generated for genes with p < 0.05 to further demonstrate their prognostic significance. Then, UMAP plots were utilized to visually describe the expression distribution of model genes across cellular subtypes, while violin plots were used to further illustrate the gene expression patterns among distinct cellular subgroups.

### Construction and validation of the nomogram model

Prognostic factors were incorporated with p-values < 0.05 from the results of multifactor Cox analysis and a nomogram prognostic model was constructed to predict the survival rates of patients at 1-, 3-, and 5-year intervals. The risk score of the model was defined as the sum of the product of factor scores and their corresponding coefficients, as illustrated below:

$$\text{Risk score}=\sum_{i=1}^{n}[{Score}_{{factor}_{i}}*{\beta }_{i}]$$

here, ${Score}_{{factor}_{i}}$ represents the score of the model factor, and ${\beta }_{i}$ denotes the corresponding coefficient. Calibration curves were then employed to evaluate the accuracy of the model in predicting outcomes at 1-, 3-, and 5-year intervals. Patients were stratified into high- and low-risk groups according to the median score. KM analysis was conducted to assess the prognosis of these groups, and receiver operating characteristic (ROC) curve analysis was performed to assess the performance of the model at the specified time points. Cumulative risk factor plots were generated to elucidate the relationship among scores, gene expression, and survival prognosis. Finally, curves for decline curve analysis (DCA) were constructed for both the nomogram model and its individual factors at 1-, 3-, and 5-year intervals to validate the advantages associated with clinical decision-making by the nomogram model.

### Weighted gene co-expression analysis and functional enrichment analysis

Weighted Gene Co-expression Analysis (WGCNA) was conducted on all genes from patient samples in the TCGA-HCC dataset, using parameters set as cutline = 100,000 and a soft threshold of 6. The analysis was visualized with a Cluster Dendrogram, and the correlation between various gene modules and the risk score was visualized by generating a heatmap. The module with the most significantly positive correlation was selected and genes with a correlation coefficient > 0.4 were identified. Subsequently, KEGG and GO enrichment analyses were performed on this gene set, and the results were visualized with chord diagrams.

### Immunological analysis

The status of immune cell infiltration in patient samples was assessed using seven immune cell infiltration prediction algorithms, including CIBERSORT-ABS, XCELL, QUANTISEQ, TIMER, MCPCOUNTER, CIBERSORT, EPIC. The Spearman correlation between the predicted immune cell scores were generated by the seven immune cell infiltration prediction algorithms and the risk score. Further, we visualized scatter plots for the top four cell categories with both the highest and lowest correlations with the risk score for enhanced visual representation.

### Gene clinical feature analysis of the nomogram model and drug prediction

We analyzed the differences in gene expression included in the nomogram model across clinical pathological factors (T, N, M, normal vs. tumor). Subsequently, we conducted a drug sensitivity analysis for each gene in the nomogram model using the Cmap database. We selected the drug with the lowest score for each gene, performed molecular docking, and displayed the successfully docked results using PyMOL. In molecular docking analysis, we examined the amino acids interacting with the small molecule drugs and determined the binding energy of the resultant hydrogen bonds. We have also experimentally determined the structure of the amino acid, with its PDB ID being P6286.

### Statistical analysis

All statistical analyses were performed using R software (version 4.1.3). Cox regression analysis was conducted employing the “survival” and “survminer” packages. Differential expression analysis was implemented for the “limma” package, and the “ggplot2” package was used for visualization. A significance threshold of p < 0.05 was considered statistically significant (*p < 0.05; **p < 0.01; ***p < 0.001; ****p < 0.0001).

## Results

### Single-cell sequencing analysis

**Fig. 1:**
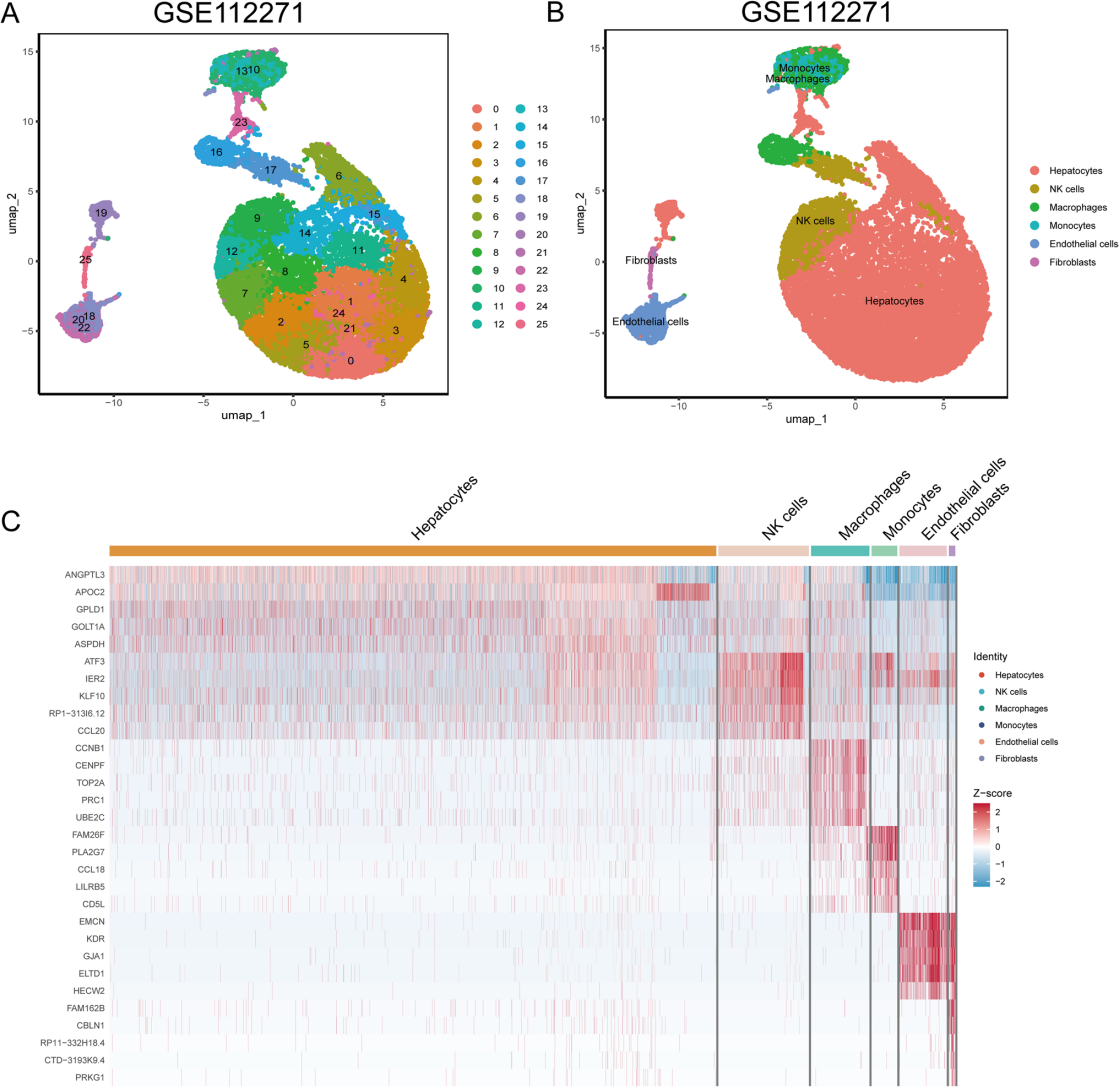
Single-cell sequencing data analysis. **A** Dimensionality reduction clustering of single-cell sequencing data GSE112271 through uniform manifold approximation and projection. **B** Cell subgroups were classified into six categories according to specific marker genes. **C** Heatmap showing specific marker gene expression for each cell subgroup

Subsequent to UMAP dimensionality reduction on the single-cell sequencing data GSE112271, the cells were annotated into six categories based on the specific marker genes of each cell subtype, including hepatocytes, natural killer (NK) cells, macrophages, monocytes, endothelial cells, and fibroblasts (Fig. 1A, B). A heatmap was generated to illustrate the expression levels of specific marker genes for each cell subtype. Genes such as ANGPL3, APOC2, GPLD1, GOLT1A, and ASPDH exhibited higher expression than other genes in the hepatocyte subtype. In the NK cells, we observed an elevated expression of ATF3, IER2, KLF10, RP1-313I6.12, and CCL20. Macrophages exhibited higher expression of CCNB1, CENPF, TOP2A, PRC1, and UBE2C. Monocytes showed increased expression of FAM26F, PLA2G7, CCL18, LILRB5, and CD5L, while endothelial cells demonstrated higher expression of EMCN, KDR, GJA1, ELTD1, and HECW2. In the fibroblasts, higher expression of genes, such as FAM162B, CBLN1, RP11-332H18.4, CTD-3193K9.4, and PRKG1 were noted than other subtypes (Fig. 1C).

### Selection of hepatocellular lipid metabolism-related genes

**Fig. 2:**
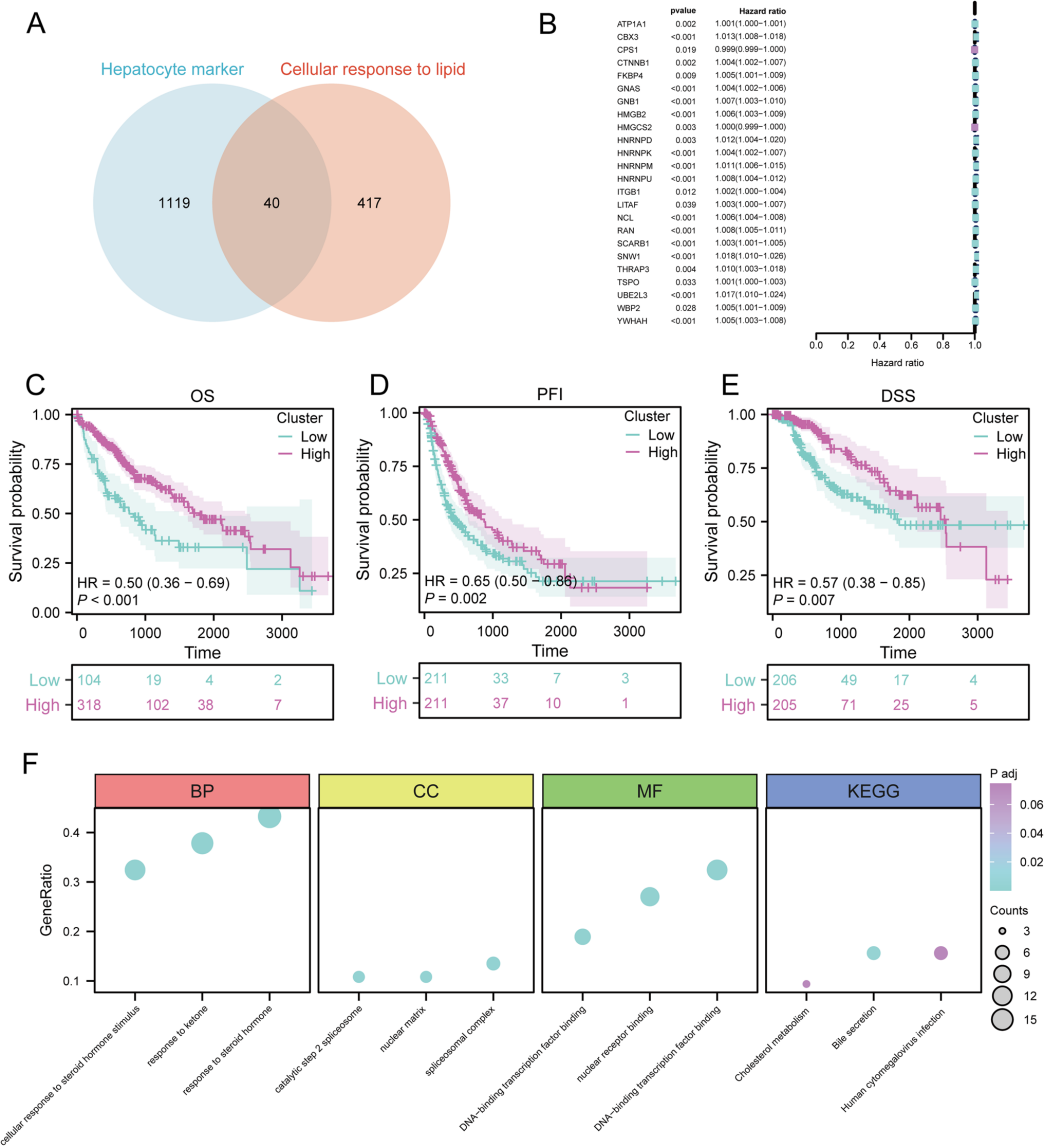
Screening of genes associated with lipid metabolism in liver cells. **A** Intersection of “cellular response to lipid” gene sets from Gene Set Enrichment Analysis (GSEA) and marker genes of the hepatocyte subgroup. **B** Univariate Cox regression analysis of intersecting genes. **C** Differences in two score groups for overall survival (OS). **D** Differences in survival among two score groups for progression-free interval (PFS). **E** Differences in survival among two score groups for disease-specific survival. **F** Visualization of Gene Ontology (GO) and Kyoto Encyclopedia of Genes and Genomes (KEGG) enrichment analysis with bubble plots

We obtained the “cellular response to lipid” gene set from GSEA and intersected this set with the marker genes of the hepatocyte subtype to identify 40 genes (Fig. 2A). Subsequently, we conducted a univariate Cox analysis to filter out 24 genes with statistical significance (Fig. 2B). We assigned comprehensive scores to these 24 genes employing the ssGSEA algorithm. The samples were stratified into high- and low-score groups based on the median score, and KM curves revealed a time-dependent decrease in survival rates for both groups. The low-score group had significantly worse OS, PFI, and DSS than the high-score group (p < 0.01). These results indicated the significance of the selected 24 genes in prognosis (Fig. 2C–E).

Subsequently, GO and KEGG enrichment analyses were performed on these 24 genes. The GO analysis indicated significant enrichment of pathways involved in cellular response to steroid hormone stimulus, response to ketone, response to steroid hormone, and DNA- transcription factor binding (Fig. 2F). The KEGG analysis showed the predominant enrichment of pathways associated with bile secretion (Fig. 2F).

### Construction and validation of machine learning prognostic model

**Fig. 3:**
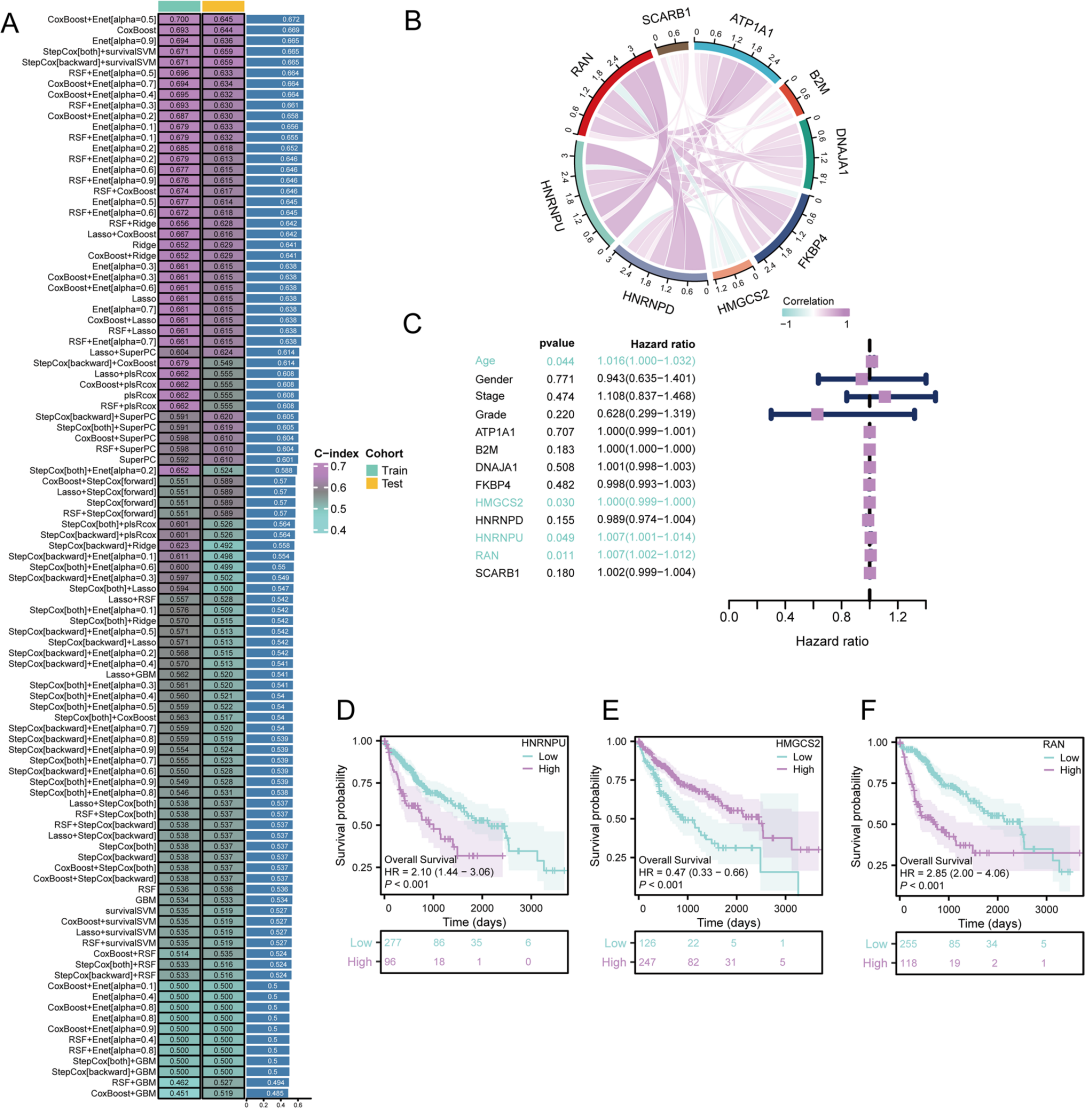
Construction of the machine learning prognostic model. **A** Prediction based on the combination of 101 machine learning algorithms. **B** Correlation of nine model genes with The Cancer Genome Atlas (TCGA)-Hepatocellular Carcinoma expression. **C** Multifactorial Cox analysis combining clinical factors. **D** Differences in survival between the two groups in terms of the expression of HNRNPU. **E** Differences in survival between the two groups in terms of the expression of HMGCS2. **F** Differences in survival between the two groups in terms of the expression of RAN

The TCGA-HCC dataset was randomly classified into equal-sized training and testing cohorts. We calculated the average C-index and C-index for both the training and validation datasets to evaluate the predictive efficacy of the models for every algorithmic combination. Among all predictive models, those employing the Enet combination demonstrated superior average C-indices consistently (C-index > 0.6). Notably, the CoxBoost + Enet [alpha = 0.5] model showed the highest training set C-index (C-index = 0.700) and average C-index (C-index = 0.672). The validation set C-index also demonstrated satisfactory performance (C-index = 0.645), hence, the CoxBoost + Enet [alpha = 0.5] algorithmic combination was selected for constructing the prognostic model (Fig. 3A).

**Fig. 4:**
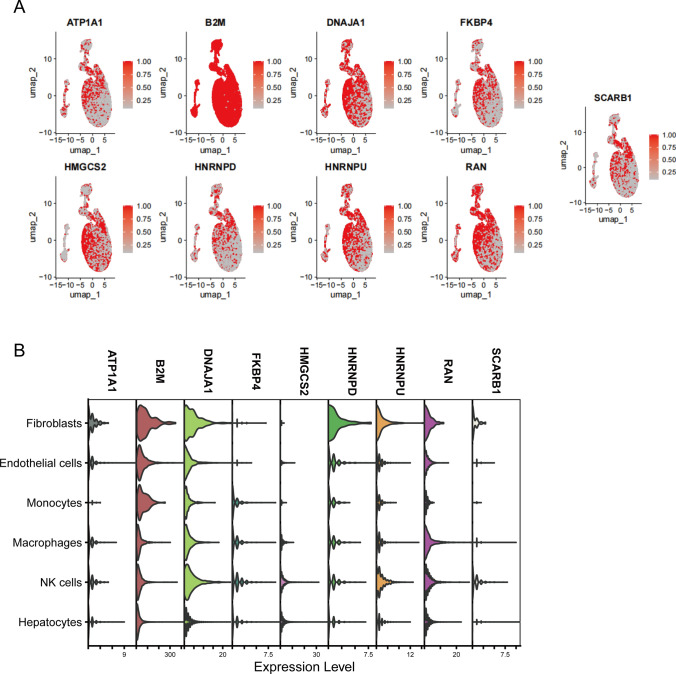
Analysis of each cell subgroup for the expression of model genes. **A** Uniform manifold approximation and projection visualization for differences in expression of model genes in each cell subgroup. **B** Violin plots showing the differences in expression of model genes in each cell subgroup

We narrowed down the initial set of 24 genes to a final set of 9 genes, including RAN, SCARB1, ATP1A1, B2M, DNAJA1, FKBP4, HMGCS2, HNRNPD, and HNRNPU using the CoxBoost + Enet [alpha = 0.5] combination. Most of these genes were positively correlated with their expressions, except HMGCS2 which showed a negative correlation with other genes (Fig. 3B). We then employed a multifactorial Cox analysis combining these nine genes with clinical features (age, gender, stage, grade) and identified four independent prognostic factors: age, HMGCS2, HNRNPU, and RAN (p < 0.05) (Fig. 3C). Subsequently, patients were categorized into high- and low-expression groups based on the median expression levels of these three genes.KM survival analyses were conducted that showed that both high and low-expression groups exhibited a decline in survival rates over time. Patients with high levels of HNRNPU and RAN had significantly poorer prognosis (p < 0.001), while low expression of HMGCS2 correlated with worse prognosis (p < 0.001). These results further reinforced the identification of HNRNPU and RAN as prognostic risk factors (HR > 2) and HMGCS2 as a prognostic protective factor (HR < 1, Fig. 3D–F). We performed single-cell sequencing analysis using the GSE112271 dataset to validate these findings. The UMAP plot was used to illustrate the expression distribution of the nine model genes across different cell subtypes. Specifically, B2M exhibited significantly high expression in all cell subtypes, while other genes demonstrated relatively high expression levels across various subtypes (Fig. 4A). Further analysis employing violin plots presented the detailed expression patterns of these nine genes in different cell subtypes. Notably, B2M, DNAJA1, HNRNPD, and HNRNPU exhibited significantly elevated expression in fibroblasts. There was also a consistently high expression of B2M and DNAJA1 across various cell subtypes. Further, the expression of HNRNPD was moderate in other subtypes, but its expression was notably high in fibroblasts (Fig. 4B).

### Construction and validation of the nomogram model

**Fig. 5:**
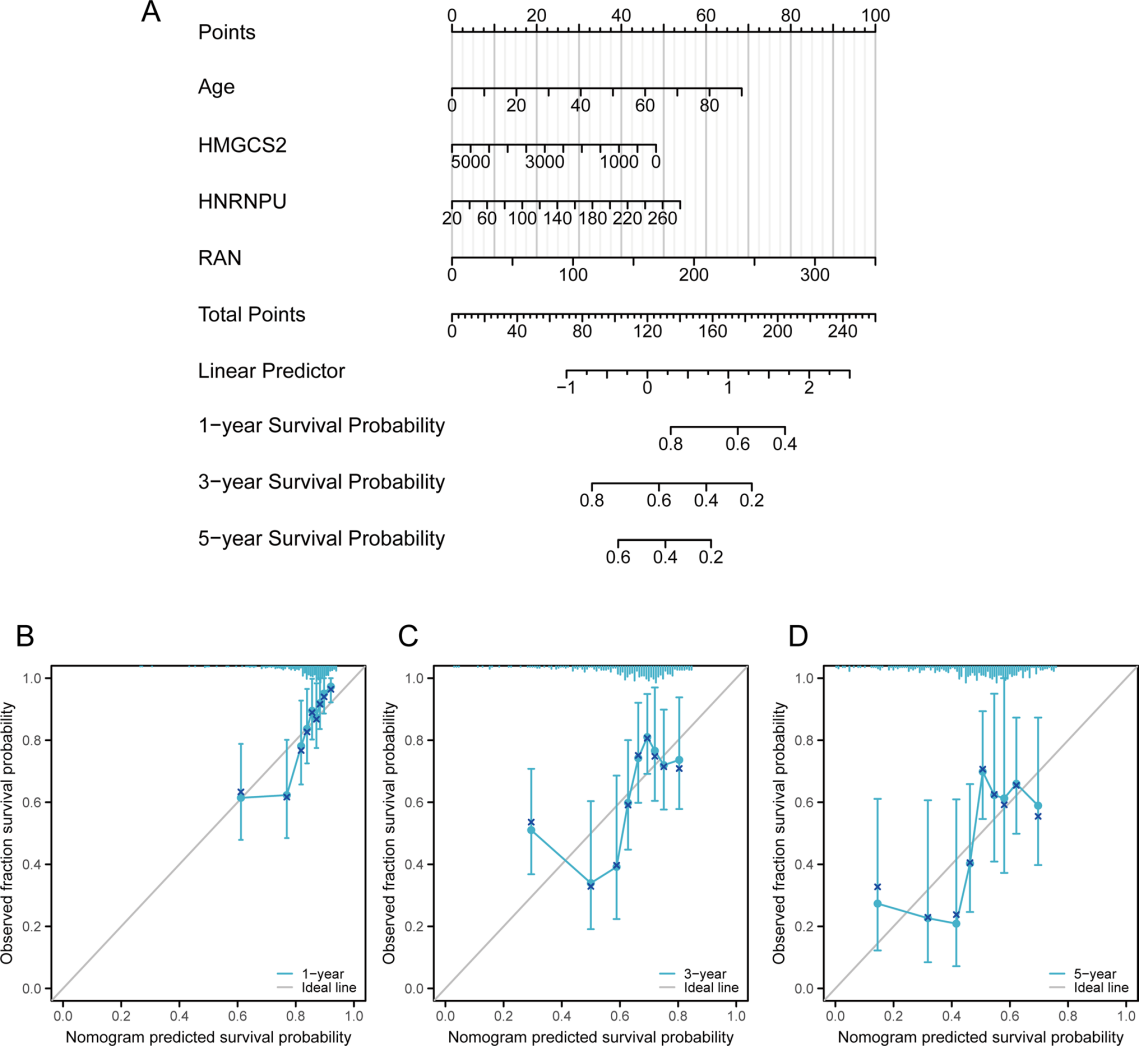
Construction and validation of the nomogram model. **A** Construction of the nomogram prognostic model. Calibration curve assessing the accuracy of the nomogram model at **(B)** 1 year and** (C)** at 3 years. **(D)** Calibration curve testing the accuracy of the nomogram model at 5 years

The prognostic factors with p-values < 0.05 from the multivariable Cox analysis (age, HMGCS2, HNRNPU, RAN) were used to construct a nomogram prognostic model to predict the 1-, 3-, and 5-year survival rates of patients (Fig. 5A). The formula used in the model was as follows:

$${\text{Riskscore}} = {\text{Age}}*0.0{153} + {\text{ HMGCS2}}* - 0.000{2} + {\text{ HNRNPU}}*0.00{42} + {\text{RAN}}*0.00{57}$$

**Fig. 6:**
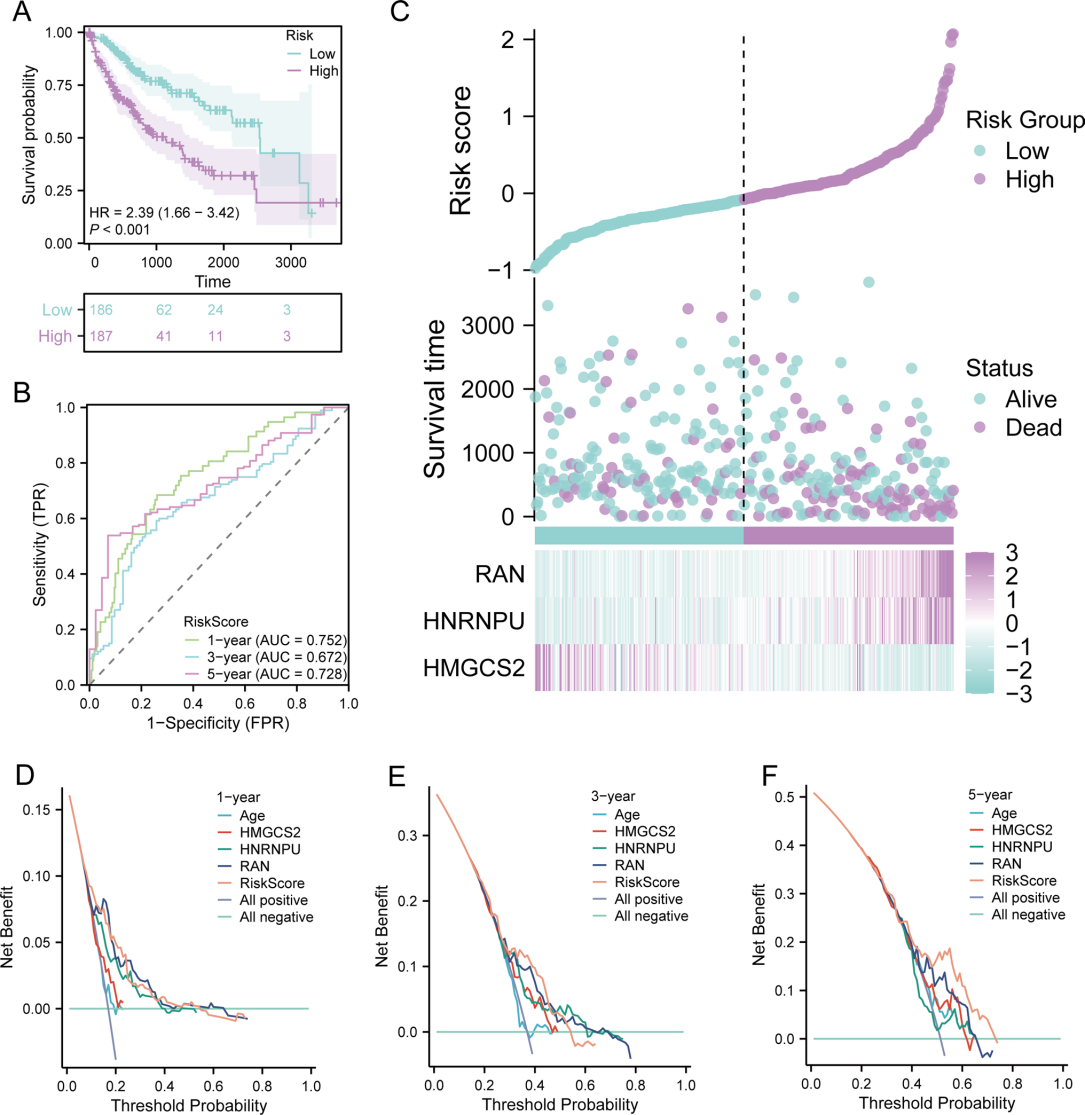
Validation of the nomogram model. **A** Differences in survival among two risk groups. **B** Receiver Operating Characteristics (ROC) curve to test the efficiency of the model at 1, 3, and 5 years. **C** Cumulative risk factor plot exhibiting the relationship among gene expression, risk scores, and survival prognosis. Decline curve analysis (DCA) of the nomogram model and associated factors **(D)** at 1 year and **(E)** at 3 years. **(F)** DCA curve of the nomogram model and associated factors at 5 years

The precision of the nomogram model in forecasting outcomes was evaluated using calibration curves over 1-, 3-, and 5-years. Our model accurately predicted outcomes at these time points, with particularly significant accuracy in short-term predictions at 1-year (Fig. 5B–D). KM survival analysis predicting the prognosis of high and low-risk groups showed that the survival rates of both risk groups decreased with time, and significantly poorer outcomes were observed in the high-risk group (p < 0.001). The nomogram risk model emerged as a risk factor for prognosis (HR = 2.39, Fig. 6A). ROC curve analysis revealed that the area under the curve (AUC) at 1, 3, and 5 years were all > 0.65, indicating the good diagnostic performance of the model at these three time points (Fig. 6B). The plot representing the risk accumulation factor illustrated a correlation between high mortality rates and increasing risk scores in both two groups, indicating a rate of survival over time. Furthermore, high expression levels of HNRNPU and RAN were observed in the high-risk cohort, while the expression of HMGCS2 was increased in the low-risk cohort (Fig. 6C). According to the nomogram model DCA at 1, 3, and 5 years, patients achieved better clinical decision-making benefits than when each factor was used individually as a decision criterion (Fig. 6D–F).

### Weighted gene co-expression network analysis and functional enrichment analysis

**Fig. 7:**
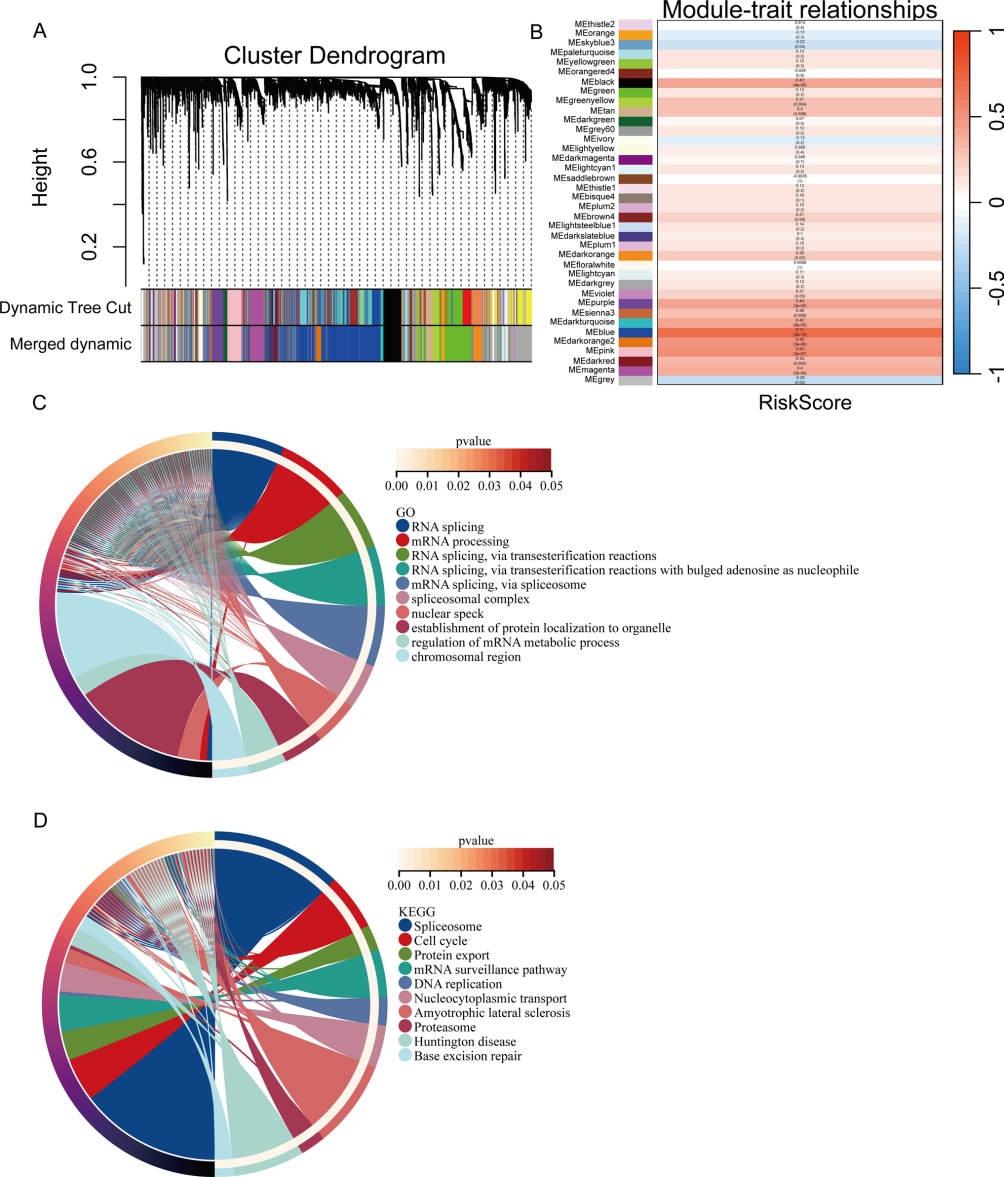
Weighted gene co-expression and functional enrichment analysis (WGCNA). **A** Visualization of WGCNA analysis with cluster dendrogram. **B** Heatmap showing the differential correlations between gene modules and risk score. **C** Chord diagram showing Gene Ontology (GO) enrichment analysis. **D** Chord diagram visualizing Kyoto Encyclopedia of Genes and Genomes (KEGG) enrichment analysis

WGCNA was conducted on all genes from patient samples in the TCGA-HCC dataset and visualized using a Cluster Dendrogram (Fig. 7A). A heatmap analysis was used to reveal the correlation between the gene modules and the risk scores. Modules such as MEblue, MEblack, MEpurple, and MEpink, showed a highly positive correlation with the risk score (R > 0.4). We selected the MEblue module with the strongest positive correlation (R = 0.71, p = 2e-14) (Fig. 7B). Genes with a correlation coefficient > 0.4 were subsequently subjected to GO and KEGG pathway analyses. GO analysis revealed significant enrichment of genes in pathways involved in RNA splicing and mRNA processing. Similarly, the KEGG analysis indicated significant enrichment of genes involved in pathways associated with nucleocytoplasmic transport and amyotrophic lateral sclerosis (Fig. 7C, D).

### Immune-related analysis

**Fig. 8:**
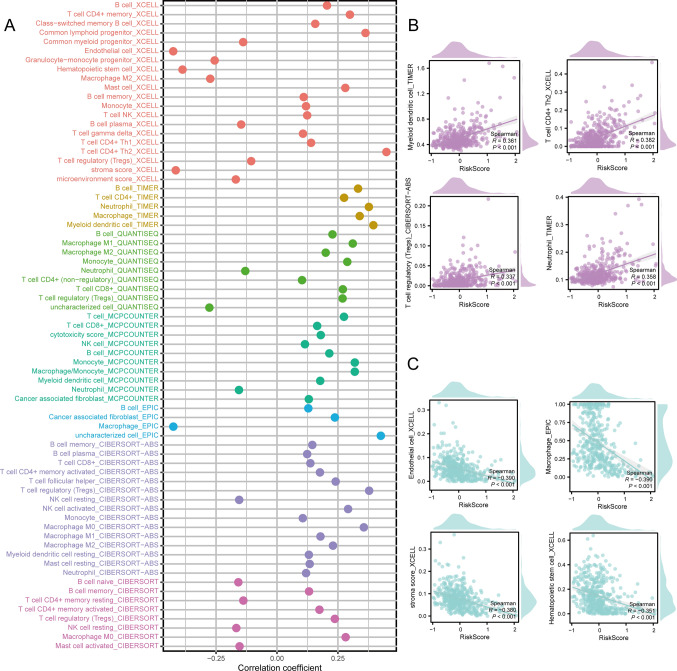
Immune-related analysis. **A** Determination of correlation between risk score and scores of various immune cells. Scatter plots showing the four cells with the **(B)** highest correlation to risk scores and **(C)** the lowest correlation to the risk score

The Spearman correlation method was used to assess the correlation between the immune cell scores predicted by seven immune analysis methods and the risk score. The seven methods used included: CIBERSORT-ABS, XCELL, MCPCOUNTER, TIMER, EPIC, QUANTISEQ, and CIBERSORT. B cells, T cell regulatory (Tregs), T cells CD8 +, and monocytes exhibited relatively high positive correlations with the risk score across multiple algorithms (Fig. 8A). Notably, myeloid dendritic cells (R = 0.361, p < 0.001), T cells CD4 + Th2 (R = 0.382, p < 0.001), T cell regulatory (Tregs) (R = 0.337, p < 0.001), and neutrophils (R = 0.358, p < 0.001) documented the highest positive correlations with the risk score. Conversely, endothelial cells (R = − 0.390, p < 0.001), macrophages (R = − 0.390, p < 0.001), stroma score (R = − 0.380, p < 0.001), and hematopoietic stem cells (R = − 0.351, p < 0.001) exhibited the highest negative correlations with the risk score (Fig. 8B, C).

### Gene clinical feature analysis of the nomogram model and drug prediction

**Fig. 9:**
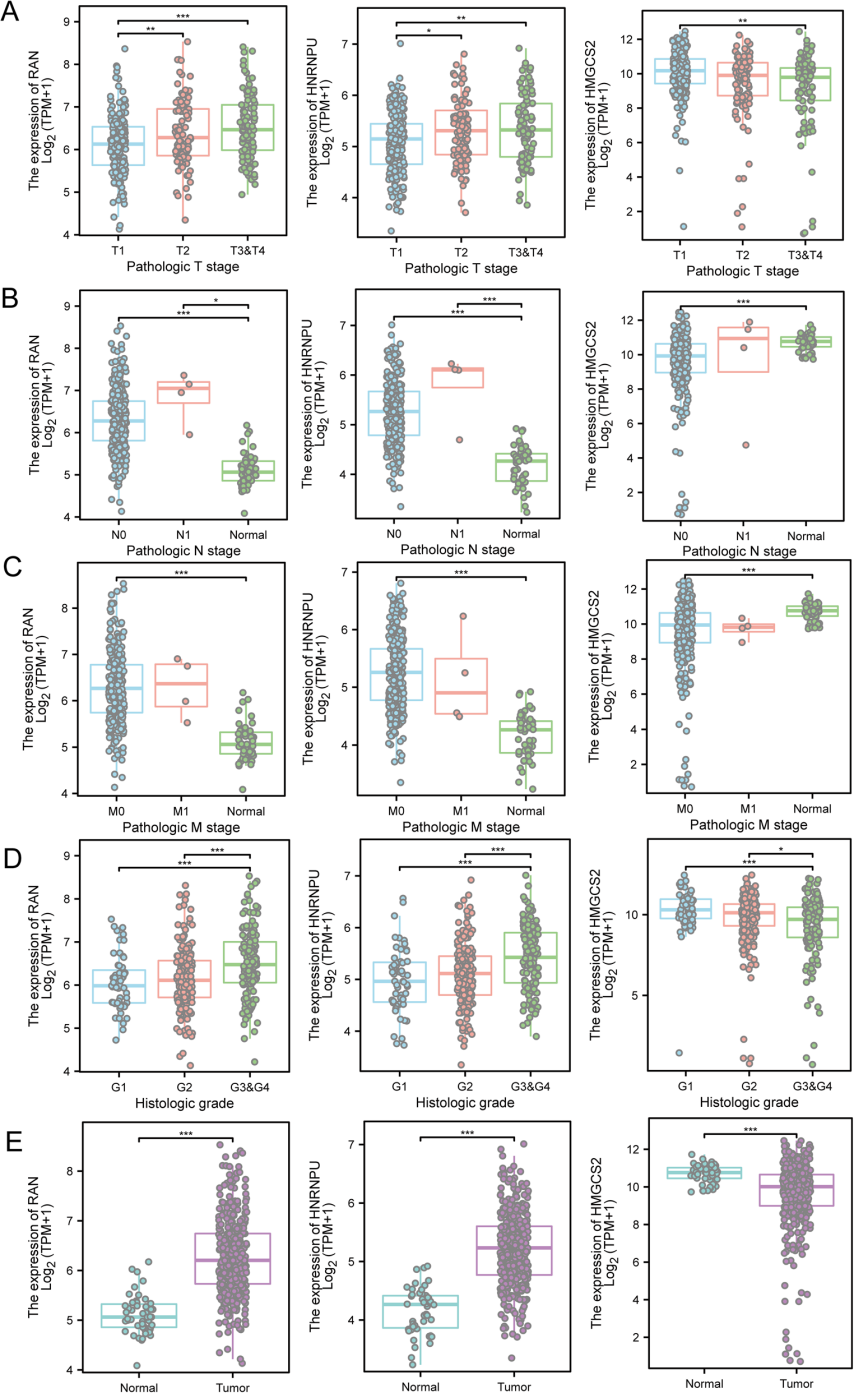
Clinical feature analysis of the genes in the nomogram model. Differential expression of RAN, HNRNPU, and HMGCS2 with regard to clinical pathological factors **(A)** clinical pathological factor T, **(B)** clinical pathological factor N, **(C)** clinical pathological factor M, **(D)** clinical pathological factor Grade, **(E)** clinical pathological factor normal vs. tumor

**Fig. 10:**
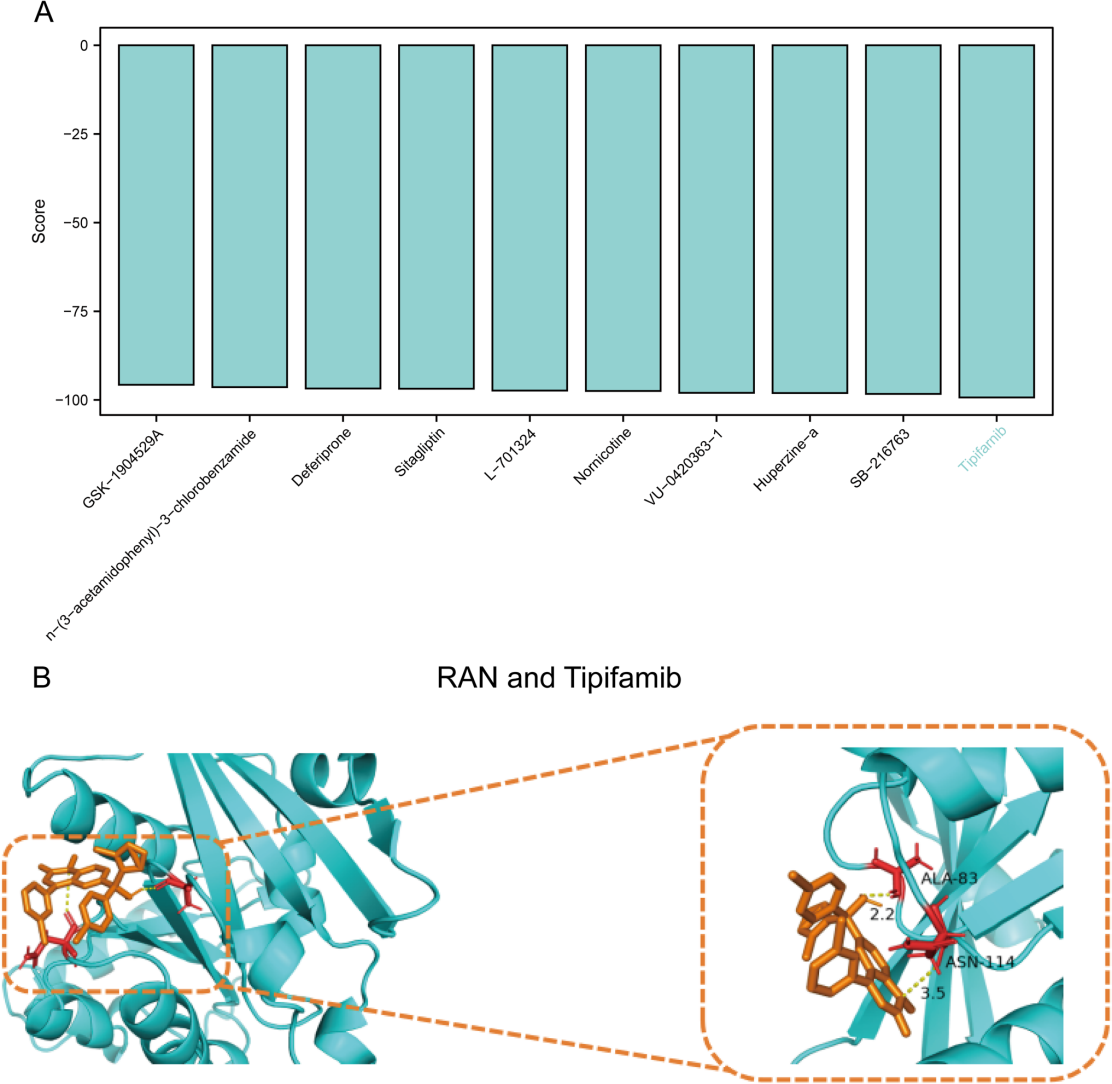
Drug prediction. **A** The scores of RAN with various drugs. **B** The results of molecular docking analysis of RAN and tipifarnib

We analyzed the differences in the expression of nomogram model genes (HMGCS2, HNRNPU, RAN) with regard to clinical pathological factors (T, N, M, normal vs. tumor). Higher expression of HNRNPU and RAN was observed in higher T stages (p < 0.05), while HMGCS2 showed lower expression in higher T stages (p < 0.01). Increased expression of HNRNPU and RAN were noted in higher N stages, while the normal tissues exhibited the lowest expression (p < 0.05). However, the opposite trend was noted for HMGCS2 in normal tissues (p < 0.001). Furthermore, HNRNPU and RAN demonstrated significantly higher expression levels (p < 0.001) while HMGCS2 exhibited significantly lower expression levels in M0 compared to normal tissues (p < 0.001). In higher grade categories, HNRNPU and RAN were expressed at higher levels (p < 0.001), while HMGCS2 exhibited lower expression (p < 0.05). The expression of HNRNPU and RAN was also enhanced in tumor tissues, with the lowest expression observed in normal tissues (p < 0.001). In contrast, the opposite trend was observed for HMGCS2 in normal tissues (p < 0.001) (Fig. 9A–E). Subsequently, we performed a drug sensitivity analysis for each gene in the nomogram model based on the Cmap database. Each gene was docked with the drug having the lowest score, of which only RAN yielded favorable molecular docking results. Subsequently, we selected tipifarnib as the drug with the lowest score (Fig. 10A, Supplymentary file 1). According to molecular docking analysis, the amino acid ALA-83 formed a hydrogen bond with the targeted drug at 2.2 Å, while ASN-114 formed a hydrogen bond with the targeted drug at 3.5 Å. Further, the binding energy was -5.9 kcal/mol, which indicated a favorable effect of the molecule on its target protein (Fig. 10B).

## Discussion

HCC is the most common form of liver cancer given its unnoticeable early progression and a dismal 5-year OS rate of < 12%. This makes the timely diagnosis challenging, leading to missed opportunities for satisfactory treatment. Advanced-stage HCC is highly invasive, for which the preferred treatment modalities include surgical resection or transplantation. However, only 20% of cases of HCC are amenable to surgical intervention, making interventional therapies necessary for patients not suitable for surgery. Although some approaches, such as transarterial chemoembolization are effective for intermediate-stage liver cancer, managing late-stage cases is challenging. Hence, investigating the mechanisms of action of HCC-related genes, constructing prognostic models, and predicting drug responses are paramount for precise treatment, early diagnosis, and improved prognosis.

We acquired matching clinical information and bulk transcriptomic data for HCC from the publicly available TCGA database, and a single-cell sequencing dataset for liver cancer (GSE112271) from the GEO database. These datasets hold immense potential for research and application to understand patient diagnosis, treatment, and prognosis. We employed UMAP dimensionality reduction for single-cell sequencing data and annotated cells into six subtypes according to specific marker genes for each cell subgroup. Subsequently, in each cell subtype, the patterns of expression of marker genes were investigated and specific marker genes for the hepatocyte subtype were identified. Intersection analysis was performed using the GSEA-derived gene set associated with the cellular response to lipids and 40 genes were identified. A univariate Cox analysis of these 40 genes yielded 24 genes, and the ssGSEA algorithm was applied to generate comprehensive scores for these genes.

We categorized the samples into high- and low-score groups based on the median score and conducted survival analysis for the OS, PFI, and DSS. There was a significant difference between the low- and high-score groups, indicating the prognostic significance of the 24 selected genes. Subsequent GO and KEGG pathway analyses of the 24 genes to explore their major enriched pathways showed the association of most of these pathways were with cellular metabolism and gene regulation.

To summarize, the integrated bulk and single-cell transcriptomic data analysis and comprehensive bioinformatics approaches identified 24 genes with prognostic significance in HCC. The enriched pathways associated with these genes offer insights into the potential molecular mechanisms underlying HCC development and progression. After randomly dividing the TCGA-HCC into training and testing cohorts with equal patient numbers, we employed various combination algorithms to determine and compare the predictive capabilities of models based on the average C-index and C-index of both cohorts. Ultimately, a predictive model was established using a combination of CoxBoost and Elastic Net (CoxBoost + Enet [alpha = 0.5]) algorithm. We identified nine genes, including RAN, SCARB1, ATP1A1, B2M, DNAJA1, FKBP4, HMGCS2, HNRNPD, and HNRNPU utilizing the CoxBoost + Enet [alpha = 0.5] method to screen 24 genes. RAN is a RAS-related nuclear protein and a small GTP-binding protein of the RAS superfamily [13]. RAN has a unique role in the regulatory pathways of macromolecular nucleocytoplasmic transport and cell cycle progression. The upregulation of RAN exerts oncogenic effects in tumors and is associated with poor prognosis. The pivotal role of the RAN protein involves facilitating RNA and protein transport across the nuclear pore complex, while regulating DNA synthesis and cell cycle progression [14]. The SCARB1 gene encodes a protein that may be a potential target in various human cancers, including HCC [15]. Cancer tissues express high levels of SCARB1, and this expression is associated with the differentiation status of liver cancer cells [16]. ATP1A1 is a member of the P-type cation transport ATPase family and the Na + /K + -ATPase subfamily, playing a crucial role in processes such as Na + /K + exchange, metabolism, and signal transduction [17]. The ATP1A1 signaling pathway is influenced by epigenetic alterations and compromised cellular autophagy and is critical in HCC oncogenesis and advancement, making it a viable target for therapeutic intervention [18]. The B2M gene encodes a serum protein closely linked with the heavy chain of the major histocompatibility complex class I, ubiquitously expressed on the surface of almost all nucleated cells. Further, B2M plays a significant biological role in tumor development and immune regulation [19]. B2M is widely expressed in most nucleated cells, such as immune cells, with stromal cells surrounding tumor cells exhibiting strong B2M expression. Previous research suggests that B2M is a promising potential target for cancer therapies in the future [20]. DNAJA1 is a member of the DNAJ/HSP40 protein family, which participates in various cellular activities, such as folding, translocation, and degradation of proteins [21]. Enhanced expression of DNAJA1 in liver cancer tissues correlates strongly with unfavorable prognosis, highlighting its oncogenic function in liver cancer cell proliferation and metastasis [22]. The FKBP4 protein is a member of the immunophilin protein family that participates in immune regulation and fundamental cellular processes involving protein folding and transportation. FKBP4 functions as an HSP90-associated co-chaperone and is a tumor-specific antigen to trigger immune responses [23]. Furthermore, FKBP4 has multiple functions in various cancers, such as lung and prostate cancers, and glioblastoma [24]. The HMGCS2 protein is a member of the HMG-CoA synthase family that functions as a key mitochondrial enzyme catalyzing the initial step of ketogenesis. making it a rate-limiting factor in this process [25]. The expression of HMGCS2 in cancer varies; the modulation of ketone production through HMGCS2 in HCC is a potential therapeutic strategy for liver cancer [26]. HNRNPD, also known as AUF1, is a hnRNP subfamily member, and is ubiquitously expressed. It is an AU-rich element-binding protein that regulates the stability of mRNAs coding for proteins involved in tumor initiation, cell cycle regulation, aging, proliferation, and apoptosis [27]. Additionally, AUF1 plays a significant role in HCC development and serves as a potential target for HCC treatment [28]. HNRNPU, also known as SAF-A, is highly important for gene transcription, post-transcriptional RNA processing, and regulation of higher-order chromatin structures [29]. Further, HNRNPU expression is upregulated in HCC, and its inhibition in vitro as well as in vivo leads to suppressed proliferation of HCC cells. Research shows that upregulated HNRNPU expression is associated with poor prognosis in HCC [30]. The expression of most genes shows positive correlations, while HMGCS2 has negative correlations with other genes. Therefore, model genes exhibiting positive correlation may serve as immunotherapeutic targets for HCC, with HMGCS2 as a prognostic protective factor; nonetheless, further experimental validation of our hypothesis is required. Multivariate Cox analysis of these nine genes combined with four clinical traits: age, gender, stage, and grade resulted in four independent prognostic factors: age, HMGCS2, HNRNPU, and RAN. Subsequently, we categorized them into two expression groups for KM survival analysis based on the median expression values of these three genes. With time, the survival rates of both expression groups for the three genes decreased, specifically, patients with high HNRNPU and RAN expression had poorer prognoses. In contrast, those with low HMGCS2 expression exhibited worse outcomes, further indicating HNRNPU and RAN as prognostic risk factors, and HMGCS2 as a prognostic protective factor. We conducted single-cell sequencing analysis on the dataset GSE112271 and generated UMAP plots to report high expression of all nine model genes across various cell subpopulations, with significantly upregulated levels of B2M in all subgroups. Violin plots revealed predominantly high expression of each model gene in different cell subpopulations, further validating the predictive ability of our model.

We included prognostic factors (age, HMGCS2, HNRNPU, RAN) with p < 0.05 from the multivariate Cox analysis results and constructed a prognostic nomogram model to predict 1, 3, and 5-year survival rates of the patients. Subsequently, the accuracy of our model was demonstrated by employing calibration curves, with the short-term prognostic accuracy at 1 year being particularly significant. KM survival analysis predicting the prognosis of the two risk groups revealed decreasing survival rates over time for both groups, and significantly worse prognoses were observed for patients in the high-risk group, thus validating our hypothesis. Further ROC curve analysis indicated good performance of the model at 1, 3, and 5 years. Risk accumulation factor plots demonstrated that with increasing risk scores, there were fewer patients with longer survival times in both two risk groups. Additionally, the high expression of HNRNPU and RAN in the high-risk group, and enhanced expression of HMGCS2 in the low-risk group, further validated our hypothesis that HNRNPU and RAN are prognostic risk factors, while HMGCS2 is a prognostic protective factor. DCA curves for 1, 3, and 5-year intervals indicated that patients categorized on the nomogram model would benefit better from clinical decisions, surpassing those obtained when each factor was considered independently as a decision criterion.

We employed WGCNA on all genes to analyze the patient samples from the TCGA-HCC dataset. Most gene modules exhibited a strong positive correlation with the risk score. We selected genes from the MEblue module with a correlation > 0.4, and conducted GO and KEGG analyses to identify pathways enriched with these genes. The identified pathways included various biological processes, such as RNA processing, protein synthesis, DNA repair, cell cycle regulation, and transport of materials between the cell nucleus and cytoplasm. Furthermore, an immune-related analysis was performed with seven immune infiltration algorithms to examine the immune cell infiltration patterns in patients, and the correlation between the immune cell scores predicted by the seven immune algorithms and the risk score was evaluated through Spearman correlation analysis. We mainly noted positive correlations, with T cell regulatory (Tregs), Myeloid dendritic cells, T cell CD4 + Th2, and neutrophils exhibit particularly significant associations; in contrast, endothelial cells, macrophages, stroma score, and hematopoietic stem cells exhibit a markedly negative correlation with the risk score. We also analyzed the differences in the expression of nomogram model genes (HMGCS2, HNRNPU, RAN) across clinical pathological factors (T, N, M staging, normal vs. tumor). Subsequently, drug sensitivity was analyzed on the model genes based on the Cmap database and the drug with the lowest connectivity score for each gene was selected to conduct molecular docking. The most promising molecular docking outcome was obtained for RAN, while drug sensitivity analysis revealed tipifarnib as the drug with the lowest score. Molecular docking analysis revealed that the drug forms a 2.2 Å hydrogen bond with the amino acid ALA-83, and a 3.5 Å hydrogen bond with ASN-114 with a binding energy of − 5.9 kcal/mol. These findings highlight the favorable efficacy of the drug towards the targeting protein. Given these findings, tipifarnib emerges as a potential and promising targeted therapy for HCC treatment.

## Conclusion

In this study, we analyzed the expression patterns of subgroup marker genes associated with HCC with data from TCGA and GEO databases. We identified genes associated with lipid metabolism in the liver, constructed a machine-learning prognostic model, and validated it. We selected model genes combined with clinical features employing multifactorial Cox analysis, and constructed and validated the nomogram model. Our models demonstrated good discriminative performance, accuracy, and clinical utility. We also performed weighted gene co-expression analysis, along with GO and KEGG functional enrichment analysis. The expression patterns of the nomogram model genes with clinical pathological factors were examined. Finally, drug sensitivity analysis was conducted to offer insights into the selection of targeted therapy. This study also has a few limitations. First, our results relied on public data sources, which limited the applicability of the results or specific conditions. Second, the mechanisms of action of these target genes and their effects on relevant HCC cell lines should be verified using molecular studies in the future.

## Data Availability

These data were derived from the following resources available in the public domain: https://portal.gdc.cancer.gov/.
